# The involvement of the aorta by cardiac magnetic resonance in the inflammatory process of acute coronary syndrome

**DOI:** 10.1186/1532-429X-11-S1-P256

**Published:** 2009-01-28

**Authors:** Geetha P Bhumireddy, John F Heitner, Peter J Cawley, Igor Klem, Manesh Patel, Anna Lisa Crowley, Jonathan W Weinsaft, Michael Elliott, Michele Parker, Robert M Judd, Raymond J Kim

**Affiliations:** 1 New York Methodist Hospital, Brooklyn, NY USA; 2 Duke Univ Medical Center, Durham, NC USA

**Keywords:** Emergency Department, Chest Pain, Acute Coronary Syndrome, Cardiac Magnetic Resonance, Aortic Wall

## Introduction

In patients presenting with acute coronary syndrome (ACS), it has been shown that multiple coronary arteries are involved during the inflammatory process. Growing literature has also revealed that carotid and aortic vessels maybe involved in the inflammatory cascade of ACS.

## Purpose

To assess the involvement of the aorta during ACS by cardiac magnetic resonance (CMR).

## Methods

We prospectively evaluated 78 patients who presented to the emergency department (ED) with chest pain and were classified as either: 1) ACS: if they had positive Troponin levels and typical chest pain or 2) Non-cardiac chest pain: if they had negative Troponins and a normal stress test or catheterization. We compared these 2 groups to a control group of 45 asymptomatic diabetic patients. The descending aortic wall area (AWA) and aortic wall thickness (AWT) were measured using a double inversion recovery T-2 weighted spin echo sequence by CMR with computer software analysis. See Table 1.

**Table 1:** Clinical and aortic wall characteristics of the study population

Characteristics	Acute coronary syndrome n = 27	Non-cardiac chest pain (ED) n = 51	Asymptomatic diabetes (Control group) n = 45	p-value
Age (yrs)	60.3 ± 11.4	53.6 ± 10.7	59.9 ± 8	
Gender (female)	52.2%	47%	44.5%	
BMI	29.19	19.85	14.10	
**Past medical history:**				
Smoker	43.5%	13.7%	11.1%	
Hypertension	78.2%	49%	75.5%	
Diabetes Type 2	21.7%	17.7%	100%	
Hyperlipidemia	30.4%	37.3%	73.3%	
**Family history of**:				
CAD	34.8%	39.2%	33.3%	
Angina	91.3%	53%	0	
MI	4.3%	0	0	
Coronary angiography	4.3%	5.9%	0	
Claudication	34.8%	0	6.7%	
Renal disease	8.7%	3.9%	0	
CVA/TIA	13%	7.8%	6.7%	
**Blood tests**:				
Troponin	1.37 ± 2.1	NA	NA	
CKMB	61.9 ± 71.1	NA	NA	
Creatinine	1.1 ± 0.33	1.1 ± 0.9	1.09 ± 0.8	
LDL	109 ± 46.2	104 ± 36.6	107.8 ± 36.3	
HDL	47 ± 11.8	47.5 ± 16.2	50.04 ± 11.8	
Triglycerides	149.8 ± 101.7	149.7 ± 76.2	212.5 ± 143	
Total cholesterol	181.8 ± 51.3	183.9 ± 43	198.25 ± 41	
CRP	2.16 ± 1.8	0.51 ± 0.6	0.61 ± 0.9	
**Medications**:				
Aspirin	21.8%	33.3%	62.2%	
Plavix	0	0	0	
Beta blockers	4.3%	17.6%	20%	
ACEI	34.8%	23.5%	55.5%	
ARB	0	0	17.8%	
Statin	43.4%	27.5%	53.5%	
Diuretics	26.1%	13.7%	-	
**Aortic wall characteristics:**				
Wall thickness (mm)				
Mean	3.173 ± 0.2	2.576 ± 0.1	2.305 ± 0.13	
Min-Max	2.0–5.4	1.1–4.0	1.2–4.2	<0.001
Wall area (mm^2^)				
Mean	2.114 ± 0.2	1.526 ± 0.06	1.382 ± 0.06	
Min-Max	1.17–4.3	0.77–2.7	0.70–1.99	<0.001
Lumen area (mm^2^)				
Mean	4.501 ± 0.248	4.3076 ± 0.136	4.006 ± 0.136	
Min-Max	2.36–8.22	2.69–8.2	1.58–7.22	0.136

Data are mean (SD) or n (percentage).

## Results

The AWA and AWT were significantly greater in patients who presented to the ED with ACS (AWA-mean 2.28 ± 0.78 mm^2^; AWT-mean 3.30 ± 0.88 mm) then both patients presenting with non-cardiac CP (AWA-mean 1.52 ± 0.39 mm^2^, p < 0.01; AWT-mean 2.53 ± 0.64 mm, p < 0.01) and the controls (AWA-mean 1.39 ± 0.36 mm^2^, p < 0.01; AWT-mean 2.36 ± 0.80 mm, p < 0.01). There was no significant difference in the patients with non-cardiac CP compared to the controls.

## Conclusion

Aortic wall may be involved in the inflammatory process of patients presenting with ACS.

